# Impact of Parental Education and Physical Activity on the Long-Term Development of the Physical Fitness of Primary School Children: An Observational Study

**DOI:** 10.3390/ijerph18168736

**Published:** 2021-08-19

**Authors:** Gerhard Ruedl, Martin Niedermeier, Lukas Wimmer, Vivien Ploner, Elena Pocecco, Armando Cocca, Klaus Greier

**Affiliations:** 1 Department of Sport Science, University of Innsbruck, Fürstenweg 185, 6020 Innsbruck, Austria; martin.niedermeier@uibk.ac.at (M.N.); l.wimmer@tsn.at (L.W.); v.ploner@tsn.at (V.P.); Elena.Pocecco@uibk.ac.at (E.P.); armando.cocca@uibk.ac.at (A.C.); nikolaus.greier@uibk.ac.at (K.G.); 2 Private Educational College, Division of Physical Education (KPH-ES), 6422 Stams, Austria

**Keywords:** longitudinal study, mixed modelling, parental education, parental physical activity, physical fitness

## Abstract

Low physical fitness (PF) has been associated with higher risk of suffering from different diseases. The importance of PF is evident already in early ages, as children’s PF appears to be a key factor of their future PF and physical activity level. Among the variables that may have an influence on children’s PF, the importance of parent’s socioeconomic status and active/inactive behaviors has been stressed in several previous studies. However, previous literature has mostly reported this association through cross-sectional studies. The purpose of this study was to examine the impact of parental education and self-reported parental physical activity (PA) on their children’s development of PF during the 4-year duration of primary education. Using German Motor Test 6-18, the major components of PF (sprint velocity, coordination, flexibility, strength endurance, power, and endurance) were measured on a total of 371 children (46.9% girls, 30.6% migration background, 19.6% overweight/obese at the fourth test time point, compliance 70.1%) from 20 primary schools in Tyrol, Austria. Results showed that children with at least one parent with upper secondary education or above obtained significantly higher PF scores at all time points compared to children with both parents with lower secondary education and below. However, PF in both groups developed over time in a comparable manner irrespective of parental education. From the age of 9 years old, children with regularly physically active parents showed a stronger development of PF over the time compared to their peers with parents reporting irregular/no PA. Our results suggest that low-educated parents’ children might be considered a special target group for interventions aiming at increasing PF. More research is needed in order to delve into the potential underdevelopment of PF in 9-year-old children whose parents have low PA levels.

## 1. Introduction

Physical inactivity in children and adolescents is considered one of the main public health problems of the twenty-first century [1], even more in past months during the COVID-19 virus lockdown [2]. A recent study by Moore et al. [2] found that only 4.8% of children (5–11 years) and 0.6% of adolescents (12–17 years) met the recommended Canadian 24-Hour Movement Guidelines during the COVID-19 outbreak. Canadian children and adolescents showed lower physical activity (PA) level, less outside time, higher sedentary behavior, and more sleep during the COVID-19 outbreak [2]. In children and adolescents, physical inactivity might lead to a reduced physical fitness [3,4] and might increase the prevalence of overweight and obesity in both developed and in developing countries [5,6]. 

There is evidence that parents influence PA and sedentary behavior of their children through role modeling (being active themselves), material support (financial, logistic, co-participation), and encouragement [7,8,9]. Moore et al. [2] reported that during the first COVID-19 virus lockdown parental encouragement and support, parental engagement in PA, and family dog ownership were positively associated with healthy active behaviors in children and adolescents. Children’s perception of parental support also plays an important role in promoting their PA [10]. The importance of parents’ practices and lifestyle on children’s active/inactive behaviors is also highlighted indirectly by other factors. For instance, previous studies have shown a correlation between their body mass index (BMI) values [11,12,13], i.e., children with an obese parent are at higher risk of being overweight or obese [14]. As a consequence, the prevalence of cardiovascular risk factors, orthopedic problems, and psychosocial constraints increases in their adult life [5,15], at the same time as there is a higher risk of reduced quality of life [16] and life expectancy by several years [17], heightening the burden on the healthcare system [18].

Among the factors that may prevent the above-stated risks, physical fitness in youth has been shown to be inversely associated with current and future overweight and obesity [19,20,21]. Physical fitness is used as an umbrella term for several skill- and health-related competences, e.g., endurance, strength, speed, or coordination [22] and can be considered a multifaceted construct [23]. Utesch et al. [23] provided evidence that it is possible to assess physical fitness using multiple separate sub-tests (e.g., specifically for endurance, strength, or coordination) and to combine the outcomes of the sub-tests to a surrogate for physical fitness. High physical fitness at young age is associated with reduced risk of being overweight or obese in adolescence [24] and low physical fitness in children and adolescents is associated with adiposity, lower cardiometabolic health, and lower bone health in later life [25]. Therefore, identifying factors associated with physical fitness of children and adolescents seems of utmost importance to develop effective intervention strategies against low physical fitness.

Physical fitness is partly genetically determined, but it can also be influenced by environmental factors [26]. Among several environmental factors, there seems to be some evidence that the socio-economic status of parents influences the physical fitness of their children [27,28]. Socio-economic status comprises factors such as parental education, occupation, income of the household, rural-urban differences, and migration background [27,29]. In a cross-sectional study by Merino-De Haro et al. [28] on more than 2600 Spanish preschoolers, a higher educational level of parents was associated with lower fatness and higher musculoskeletal fitness of their children. Results from a cross-sectional study by Lämmle et al. [27] on about 2600 German children and adolescents revealed that children with migration background and with a lower socio-economic status were less active, which resulted in lower levels of physical fitness. In another cross-sectional study by Klein et al. [30] on a cohort of about 1400 German children and adolescents aged 7 to 16 years, a higher socio-economic status was associated with a higher physical fitness. More specifically, physical fitness of children and adolescents with higher socio-economic status was significantly higher compared to the physical fitness of a German reference sample, while children and adolescents with low socio-economic status did not significantly differ from such reference sample [30]. Recently, we showed in a longitudinal study that primary school children with and without migration background significantly increased their physical fitness in a comparable manner from six to eight years [31]. However, children with migration background had significantly lower physical fitness at all test time points, which was only partly explained by a higher mean BMI in these children [31].

In addition to the socio-economic status of parents, children’s PA and physical fitness might also be affected by parental role modelling [7,8]. A nationwide Swiss study by Bringolf-Isler et al. [32] assessed the cross-sectional association between objectively (via accelerometers) measured PA of parents and their 6-to-16-year-old children. Parental moderate to vigorous PA (MVPA) was positively associated with MVPA of their children, and this correlation was strongest for children aged 10–12 years [32]. In addition, Rodrigues et al. [33] studied a cohort of more than 800 Portuguese children aged 6–10 years, finding out that parental organized and unorganized PA is positively associated with their children’s extracurricular sport participation. Compared to physically inactive parents, having physically active parents increased the odds of children participating in a greater number of extracurricular sports and more frequently, also after adjusting for age, family income, and parental education [33].

To date, the majority of the studies have focused on cross-sectional designs describing the association between children’s physical fitness and environmental factors and a possible association between parental role modelling and children’s PA. On the other hand, both evidence on parental role modelling on physical fitness and information regarding the long-term development of physical fitness in primary school children with regard to parental education and parental PA is scarce. Although the existing literature has widely confirmed that parents’ status and behaviors are linked with their children’s lifestyle, the actual impact of the former over the latter may only be ascertained by means of longitudinal approaches. For this reason, the aim of this study was to evaluate the impact of parental socioeconomic status and active behaviors on the development of their children’s physical fitness by means of a longitudinal design tracking participants during the 4 years of primary education. The objectives of this research were: (a) To assess the long-term influence of parental education on their children’s development of PF; (b) to evaluate the influence of parent’s PA on children’s changes of PF over the 4-year primary education period; and (c) to examinate differences in the association between parents’ education and PA and their children’s PF based on children’s weight status and migration background.

The following article is presented in accordance with the STROBE reporting checklist.

## 2. Materials and Methods

### 2.1. Patient and Public Involvement

There was no patient or public involvement in any of the phases of the study.

### 2.2. Sample

The present observational study was part of a larger-scaled project, where 529 children from 20 different primary schools in Tyrol, Austria, were tested over the 4 years of primary education [31]. Only those children whose parents’ questionnaire responses were available were included in the present analysis, resulting in a total of *n* = 371 (70.1%) children.

The study was conducted according to the ethical standards of the Declaration of Helsinki (as revised in 2013) and was approved by the educational board for Tyrol and the Institutional Review Board for Ethical Issues of the University of Innsbruck (19/2014). All parents received written information about the study via the participating schools and provided written informed consent before taking part in the study.

### 2.3. Measurements in Children

Height and weight were measured with children wearing sport clothes and barefoot. The height measures were taken using a mobile stadiometer “Seca 217” (Seca, Hamburg, Germany) with an accuracy of 0.1 cm and the body weight was measured with a calibrated scale “Grundig PS 2010” (Grundig AG, Neu-Isenburg, Germany) with an accuracy of 0.1 kg. Based on these data, BMI (kg/m^2^) was calculated for every child. In line with the methods described in our earlier work [31], we classified children according to their weight status into two groups: overweight (including overweight and obese children) and non-overweight (including anorexic, underweight, and normal weight children). As an indicator for migration background, based on a previous study, children were asked whether the language spoken at home was German (non-migrants) or another one (migrants) [31]. 

Physical fitness was assessed for four consecutive years (in April/May) in the same children using the German Motor Test (GMT) 6-18 [34]. The GMT 6-18 is a standardized test battery consisting of 8 items testing different subdomains of physical fitness [34]: 20-m sprint (sprint velocity), balancing backwards on three 3 m long beams with different width (coordination in a task requiring precision), jumping sideways over a middle line for 15 s (coordination under time pressure), stand-and-reach (flexibility), push-ups in a period of 40 s (strength endurance), sit-ups in a period of 40 s (strength endurance), standing long jump (power), and 6-min run (endurance). According to Bös et al. [34], the inter-rater reliability (0.95) and test-retest reliability (0.82) of the test battery were good, and the battery has been validated for assessing speed, coordination, flexibility, strength, and endurance. Combining the results of several subtests of the test battery to a single composite score for physical fitness was shown to be a valid approach [23].

Values of the test items were standardized using the means and standard deviations of the normative sample with analogous age and sex according to Bös et al. [34]. The guidelines of the test manual include multiplying the standardized score by 10 and adding 100 so to obtain a so-called Z-score, where 100 equals to average performance in the tests [34]. Z-scores above 100 mean over-average performance and Z-values below 100 under-average performance [34]. Following Utesch et al. [23], only seven subtests (20-m sprint, balancing backwards, jumping sideways, push-ups, sit-ups, standing long jump, and 6-min run) were combined into a total Z-score by using the average Z-score of the seven subtests. The eighth subtest, i.e., stand-and-reach for flexibility, was not included in the total Z-score owing to theoretical assumptions regarding the construct of physical fitness [23]. Additionally, Utesch et al. [23] suggest that the sensitivity of the subtest “balancing backwards” might not be given from the age of eight years. Therefore, a total Z-score-6 based on the average Z-scores of the remaining six subtests (20-m sprint, jumping sideways, push-ups, sit-ups, standing long jump, and 6-min run) was calculated and tested, as well.

All tests were carried out by specially trained physical education students in the sports halls at the participating schools under the exact instruction of the published test manual by Bös et al. [34].

### 2.4. Questionnaire for Parents

Parental education and parental PA (separately for both the mother and father) were collected using a questionnaire answered by the parents of the children in the second year of data collection. Parents had to specify their highest level of education (middle school, high school specializing in business, high school specializing in engineering, high school diploma, college, university, others) and their weekly PA (none, irregular, regular 1–2 times per week, regular 3 and more times per week).

Parental education was defined as 1: at least one parent completed upper secondary education (i.e., high school diploma) or above and 0: both parents completed lower secondary education or below, following categorization of each parent’s education in the same categories. The categories of parental education were selected according to the International Standard Classification of Education [35]. Similarly, parental PA was defined as 1: at least one parent regularly physically active (at least 1–2 times per week) and 0: both parents irregularly/not physically active, following categorization of each parent’s PA in the identical categories. In case of single parents, parental education and parental PA of the single parent was used. 

### 2.5. Statistical Analysis

SPSS Statistics version 25 (IBM, New York, NY, USA) was used for the analysis. The primary analysis consisted of a 2 × 2 × 4 mixed analysis of variance (ANOVA) where parental education and parental PA were used as between-subject factors and time (year 1, 2, 3, 4) as within-subject factor. The focus was put on the main effects of parental education and parental PA, as well as on parental education*time and parental PA*time interactions. Repeated contrasts were used for detailed interaction analysis since the factor time contained four stages. Main effects were interpreted as different physical fitness of children between subgroups and interactions were interpreted as different changes of physical fitness of children between subgroups over the time course of primary school. 

Additional analyses included an analysis of covariance (ANCOVA), where weight status and migration background were included as covariates to the model based on our previous findings [31]. Whenever the assumption of sphericity was violated in the ANOVA/ANCOVA (assessed by Mauchly-test), Greenhouse-Geisser correction was applied. 

Furthermore, multilevel modelling was used to check the robustness of the results of the ANOVA/ANCOVA and to account for both the hierarchical structure of the data and missing values. Following the suggestions of Field [36], full maximum-likelihood estimation method was used and different models were compared based on χ² tests on the difference in the value of minus twice the log-likelihood (−2LL). All analyses were conducted with physical fitness as the main outcome, according to the total Z-score of the seven subtests. These analyses were also run with the total Z-score-6 as the outcome. 

Weight status (overweight vs. non-overweight) and migration background (yes vs. no) were compared across parental education group and parental PA group using Pearson’s χ² tests. The distribution of parental education groups in parental PA groups was compared using Pearson’s χ² tests. Potential differences between included and excluded children in the ANOVA/ANCOVA were analyzed using independent t-tests and Pearson’s χ² tests, as appropriate. *p*-Values of less than 0.05 were considered as significant. Unless otherwise stated, values represent mean (SD) and relative (absolute frequencies).

## 3. Results

### 3.1. Parental Variables

Out of all parents, 60.4% (224) reported that at least one parent completed upper secondary education or above and in 38.3% (142), both parents completed lower secondary education or below; missing cases: 1.3% (5). At least one parent reported to be regularly physically active in 70.1% (260) and both parents reported to be irregularly/not physically active in 29.9% (111); no missing cases. The percentage of the group where at least one parent was regularly physically active was significantly higher when at least one parent completed upper secondary education (77.7 %) compared to the group where both parents completed lower secondary education or below (57.7 %), χ² (1, *n* = 366) = 16.42, *p* < 0.001.

### 3.2. Children’s Characteristics

Out of all children, 46.9% (174) were female and 53.1% (197) were male. Mean age at the four different test time points was 6.9 (0.5), 7.9 (0.6), 8.9 (0.7), and 9.9 (0.6) years, respectively. Migration background was reported in 30.6% (113) of the children; missing cases: 0.5% (2). Valid percentage of overweight or obese children at the four different test time points was 14.3%, 16.8%, 17.9%, 19.6%, respectively. Mean raw values of all physical fitness subtests improved from year 1 to year 4 with the exception of stand-and-reach where the highest values were found in the first year (Table 1).

**Figure 1:**
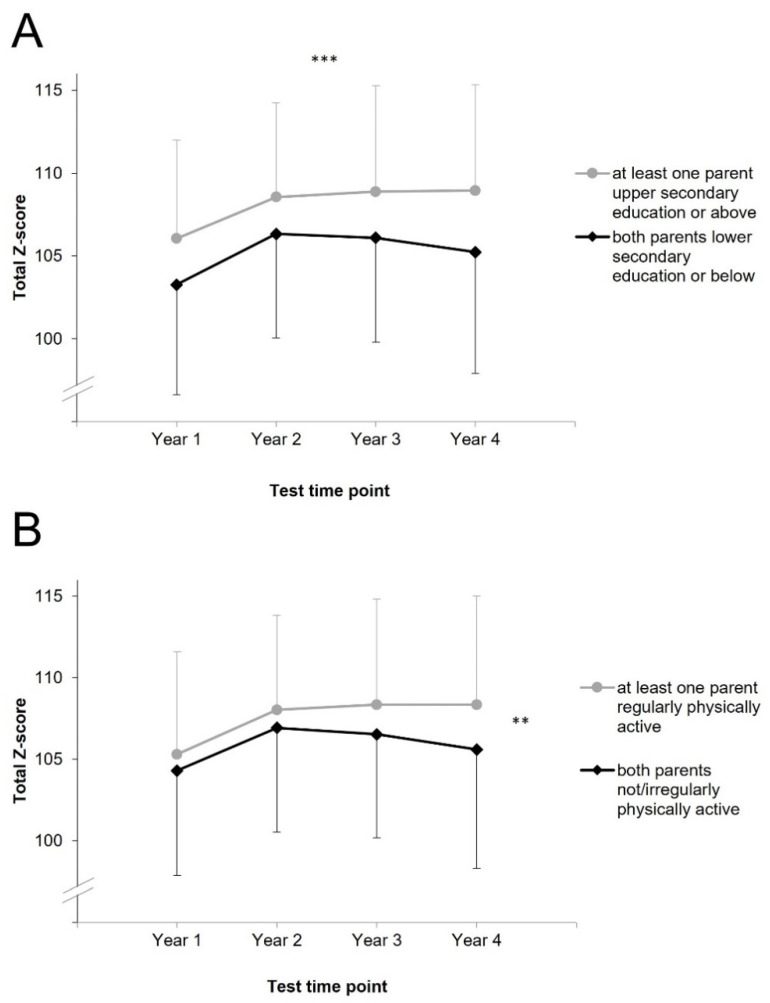
Children’s mean physical fitness values by parental education (**A**) and by parental physical activity (**B**), case wise missing. Error bars represent standard deviations. ***: indicates a significant main effect of parental education, **: indicates a significant interaction effect of parental physical activity x time according to the results of analysis of (co)variance (not significant according to multilevel modelling analysis).

Mean physical fitness of children according to total Z-scores separately for the subgroups for the four years is shown in Figure 1. A significant main effect for parental education (but not for parental PA) was found in the ANOVA (Table 2), indicating different physical fitness between children with at least one parent with upper secondary education or above and those whose both parents have lower secondary education or below. Specifically, children with at least one parent with upper secondary education or above showed higher physical fitness compared to children whose both parents did not have an upper secondary education. A significant interaction was found for parental PA*time (but not for parental education*time) indicating different changes of physical fitness between children whose both parents were not/irregularly physically active and those who had at least one parent regularly physically active. Repeated contrasts revealed the change between year three and four as statistically significant, *p* = 0.013. The change between year one and two, *p* = 0.423, and between year two and three, *p* = 0.067, did not show statistical significance.

Significant main effects were found for time, weight status, and migration background indicating an increase of physical fitness over time, higher physical fitness in non-overweight children and in children without migration background. 

The results of the ANCOVA were similar to the ANOVA showing a significant main effect of parental education and a significant interaction between parental PA*time, when weight status and migration background were included as covariates to the analysis (Table 2). Due to missing values, a total of 249 children were included in the ANOVA/ANCOVA.

For the mixed modelling analysis, six different models were calculated with similar results. Similar to the ANOVA, the primary model contained main effects of parental education, parental PA, time and interactions of parental education*time, and parental PA*time. χ² tests on the difference in the value of minus twice the log-likelihood indicated significant improvements of the models, when weight status, *p* < 0.001, and migration background, *p* < 0.001, were included. The variance in intercepts of physical fitness across children was significantly different, variance = 23.8, *p* < 0.001. When classes or schools were included to the model, no significant improvement was found, *p* > 0.283, indicating a similar variance (<0.4) in intercepts of physical fitness across classes and schools. Therefore, the final model did not consider classes and schools. The results of the final model indicated a significant main effect of parental education (Table 2). The main effect for parental PA and the interactions of parental education*time and parental PA*time were not significant. The interpretation of all analyses (ANOVA, ANCOVA, mixed modelling) was identical, when total Z-score-6 was used as the outcome instead of total Z-score-7. 

### 3.3. Comparison of Parental Education Group and Parental PA Group

The frequency of overweight children was significantly higher in children with both parents having completed lower secondary education or below compared to children who had at least one parent having completed upper secondary education or above (Table 3). No significant difference in weights status was found for parental PA groups. Percentage of migration background was significantly different across both parental education groups and parental PA groups. Higher percentage of children with migration background was found in children with both parents with lower secondary education or below and in children whose both parents were not or irregularly physically active. 

### 3.4. Comparison of Included and Excluded Children to the AN(C)OVA

The frequency of children with migration background was significantly lower in children included to the AN(C)OVA (25.7%) compared to the excluded ones (40.8%). Physical fitness according to the total Z-score in year 2, 3, and 4 was significantly higher in children included to the AN(C)OVA (108 (5.6–6.6)) compared to the excluded ones (106 (6.7–7.7)). No significant differences between included and excluded children were found for sex, overweight status, and physical fitness in year 1. 

## 4. Discussion

The aim of the present study was to evaluate the impact of parental education and parental PA on the development of the physical fitness of their children during the 4 years of primary school days. Our results revealed that children with at least one parent with upper secondary education or above showed significantly higher physical fitness scores at all test time points compared to children with both parents with lower secondary education and below. However, physical fitness in both groups increased over time in a comparable manner irrespective of parental education. Children with parents reporting to be regularly physically active showed a stronger development of physical fitness over time compared to their peers with parents reporting irregular or no PA. The diverging development of physical fitness was evident between year 3 (mean children’s age: 9 years) and year 4 (mean children’s age: 10 years). 

### 4.1. Parental Education and Children’s Physical Fitness

The finding on the importance of parental educational status with regard to the children’s physical fitness fits well to the previous literature [9,10,37]. In accordance with the present results, Zaqout et al. [37] reported higher cardiorespiratory fitness, higher strength in the lower limbs, and higher balance in boys as well as in girls aged 6–11 years with higher educated parents. In addition, Vermeiren et al. [38] found that primary school children with higher educated mothers showed higher cardiorespiratory fitness and higher handgrip strength. Higher parental education might be associated with more sports participation and a higher involvement in recreational activities and probably with a higher awareness of the health effects of PA [38]. In addition, the importance of parental educational status was similarly shown for nutritional health behavior, i.e., a higher daily consumption of fruits and vegetables and more breakfasts on a daily basis in children with higher educated parents [38]. Parental educational status is not the only variable in the socio-economic status of families. In addition to parental education, various other indicators (e.g., migration background, household income, or profession) are used to describe the socio-economic status [27,30]. Thereby, these variables may be seen as intertwined. In the present study, the percentage of children with migration background was more than two times higher in parents with lower education compared to parents of higher education (47% vs. 21%) indicating a strong relation between migration background and the level of education. However, as indicated by both the results of the ANCOVA and of the multilevel modelling analysis, the main effect of parental education was still significant, when migration background was included into the analyses. This indicates a relatively robust finding of lower physical fitness in children of parents with lower secondary education and below. Furthermore, children with migration background showed lower physical fitness compared to children without migration background. This result is well comparable to our earlier study showing that primary school children with and without migration background had a significant increase of the overall physical fitness over the time period of 2.5 years, however, with significant lower levels of physical fitness at all 5 test time points for children with migration background [30]. In addition, Lämmle et al. [27] found that children with migration background were less physically active, which subsequently resulted in lower levels of physical fitness. By comparison, Klein et al. [30] found that children and adolescents of higher socio-economic status exhibit a higher physical fitness compared with those of lower socio-economic status.

Mean physical fitness level of children with lower educated parents hardly outreached the baseline physical fitness level of children with higher educated parents in the second year. Keeping in mind that lower levels of physical fitness in young ages are associated with adiposity, lower cardiometabolic health and lower bone health in later life [25], an adequate improvement of physical fitness during primary school days seems of utmost importance. As different factors of the family’s socio-economic status seem hardly changeable, primary school is an important place to increase health-related behavior including PA and physical fitness of young children irrespective of parental education, migration background or household income. 

### 4.2. Parental Physical Activity and Children’s Physical Fitness 

No significant effect of parental PA on children’s physical fitness was found in the present study. The possibilities to compare this finding with existing literature are limited. However, previous studies reported a positive association in the PA of parents and children [32,33]. Furthermore, Zaquout et al. [37] explored determinants of physical fitness in European children aged 6–11 years, finding that children’s PA was an independent and strong determinant of their physical fitness. Therefore, we expected an effect of parental PA on children’s physical fitness. Bringolf-Isler et al. [32] compared objectively measured PA of Swiss parents and their children aged 6–16 years. They found that an increase of 1 min of mother’s and father’s MVPA was associated with 0.24 and 0.21 min more MVPA in children, respectively [33]. In line with this, in a 30-year study, a higher parental PA was systematically associated with higher PA in children and young adults until the age of 24 years [39]. However, such a difference in children with differently educated parents was not visible in the present study. One reason for this discrepancy might be found in the age of the children. Bringolf-Isler et al. [32] argued that the association in PA of parents and children was strongest for children aged 10–12 years. Consequently, the difference in physical fitness in children with differently educated parents might occur after the age of 10 years. This assumption is supported by the significant interaction in AN(C)OVA, indicating that physical fitness in children with parents reporting to be regularly physically active showed a stronger development of physical fitness over time compared to their peers with parents reporting irregular or no PA. While showing a similar development in physical fitness between year 1 and 2 (and to a lesser amount between year 2 and 3), the diverging development in physical fitness started between year 3 (mean children’s age: 9 years) and year 4 (mean children’s age: 10 years). As children spend most time of the week in the school, it seems important to implement interventions regarding improvements of pupils’ physical fitness and PA prior to the third year of primary school. Kriemler et al. [40] evaluated the effects of a one-year school-based PA program on fitness, adiposity, and PA in primary school children in Switzerland. They found that a multicomponent PA program including two additional physical education lessons per week, daily short activity breaks, as well as PA homework, improved not only PA at school and at home and weight status, but also children’s physical fitness [40].

### 4.3. Comparison of the Statistical Models

When comparing the three statistical models used, the results are widely similar. Differences between AN(C)OVA and multilevel modelling become evident in the results of parental PA*time interaction. A significant interaction of parental PA and time was found in the AN(C)OVA, but not in the mixed modelling analysis. In contrast to the AN(C)OVA, where 249 children were included due to missing values, mixed modelling takes into account the information of all 371 children, including the cases with missing values [35]. When this information was included into the analysis, the parental PA*time interaction became non-significant. Given these discrepant results, we cannot draw a final conclusion of a potential parental PA*time interaction. However, a potential detrimental effect of low parental PA over time on children’s physical fitness should be taken into consideration in both primary school settings and in future studies on the topic. Given the lower percentage of migrants and the higher physical fitness in the participating children, the consideration of a potential detrimental effect of low parental PA might be more relevant in children without migration background and with higher physical fitness. This hypothesis is supported by a mixed modelling analysis on all children without missing values, where a significant parental PA*time interaction was found (analysis not shown in the results section). 

### 4.4. Children’s Physical Fitness and Additional Associating Factors 

Our results also revealed a higher physical fitness in non-overweight children. Although a recent study suggests that differences in specific components of PF may not be sharp across BMI categories [41], our findings are well comparable with a wide body of literature [37,42]. In an earlier longitudinal study on primary school children from first to third grade, we found that overweight children showed significantly lower physical fitness at all test time points and they did not even outreach the mean baseline fitness level of non-overweight children [42]. In the present study, the frequency of overweight children was significantly lower in children with at least one parent having completed upper secondary education or above, which is in line with the outcomes from Merino-De Haro et al. [28], who reported that preschoolers aged 3–5 years whose parents had higher educational levels showed lower body fat. In addition, Furthner et al. [13] found that low parental education levels, higher parental BMI, and migration background, were associated with overweight and obesity in 10-year-old Austrian children. Having an obese parent is associated with higher risk of being overweight or obese among children [14]. Similar to parental education status, it could be speculated that higher educated parents have a higher level of awareness and knowledge regarding positive effects of maintaining a healthy body weight including both being physically active and having a healthier diet, e.g., daily fruit and vegetable consumption [38]. This conformed to our findings that higher educated parents showed a significantly higher proportion of being regularly physically active. 

## 5. Limitations and Strengths

The following limitations have to be considered when interpreting the findings: probably, the most critical point of the present study is the self-reported nature of the assessment of parental variables, which increases the risk of non-truthfully answered questions, potential recall biases, or social desirability biases. Connected to that, the assessment and categorization of both parental education and PA might be oversimplified. Regarding parental education as a sub domain of socio-economic status, other studies used a more systematic approach to assess this multi-facetted construct, e.g., Klein et al. [29]. Additionally, other authors have stressed the role of other factors, such as children’s biological characteristics, on the determination of PF [43]. Regarding PA, future studies might consider using already validated questionnaires to assess PA levels, e.g., the International Physical Activity Questionnaire (IPAQ) [44]. 

However, several strengths of the study can be mentioned: in contrast to the majority of previously conducted cross-sectional studies [27,30], we followed the children for four consecutive years. Although some children showed missing values at the different test points, this design allowed assessing intra-individual changes. Longitudinal studies provide with stronger and more reliable information on the association and cause-effect relation among variables. Since no previous study had used such approach for the evaluation of the variables included in our work, the significance of this work lays on the possibility to understand the link between parents’ status and behaviors and their children’s lifestyle in a deeper and more impacting manner. To strengthen this approach, we also used different statistical models to assess the robustness of the findings. Therefore, the present study might provide a solid basis for the identification of specific target groups for intervention studies with the aim to increase physical fitness in primary school children.

## 6. Conclusions

Parental education is associated with physical fitness among Austrian primary school children from first to fourth grade. Children with low educated parents were disadvantaged regarding their physical fitness. Because physical fitness in children and adolescents is associated with adiposity, lower cardiometabolic health, and lower bone health in later life, interventions to increase physical fitness might focus on primary school children with low educated parents. Given the mixed results on a potential detrimental effect of low parental PA on children’s physical fitness, further research is required. However, starting from the age of 9 years, children of parents with low PA might be at risk for lower development of physical fitness compared to children of parents with regular PA. Considering that physical education, and the school environment more in general, represent the only setting in which most of these children have the opportunity to exercise and learn healthy active habits, the development of school-based programs that not only focus on the immediate increase of PF, but also on motivating students to be active, is highly recommended already in primary schools [45,46].

## Figures and Tables

**Table 1:** Mean (SD) raw values of all physical fitness subtests over 4 years.

Subtest	Time Point							
	Year 1		Year 2		Year 3		Year 4	
20-m sprint [s]	4.6	(0.5)	4.3	(0.4)	4.1	(0.4)	4.0	(0.3)
Balancing backwards [n steps]	29	(10)	34	(9)	38	(8)	39	(8)
Jumping sideways [n jumps]	25	(6)	33	(6)	37	(6)	40	(7)
Stand-and-reach [cm]	1.0	(6.7)	0.8	(6.7)	0.4	(7.2)	0.8	(7.9)
Push-ups [n]	13	(4)	17	(3)	17	(4)	18	(5)
Sit ups [n]	16	(5)	20	(6)	22	(6)	24	(6)
Standing long jump [cm]	119	(18)	124	(20)	137	(20)	143	(23)
6-min run [m]	824	(247)	876	(157)	919	(171)	966	(190)
Missing cases	64–66		49		36		24	

**Table 2:** Results of analysis of variance. analysis of covariance and multilevel modelling in comparison.

	ANOVA			ANCOVA			Multilevel Modelling					
	*p*		Partial η²	*p*		Partial η²	*p*		B	SE B	95% CI lb	95% CI ub
PEd	<0.001	***	0.06	0.001	**	0.04	0.040	*	1.75	0.67	0.42	3.07
PPh	0.326		0.00	0.677		0.00	0.288		1.33	0.70	−0.05	2.71
PET	0.231		0.01	0.261		0.01	0.269		0.31–0.95	0.54–0.55		
PPT	0.003	**	0.02	0.004	**	0.02	0.122		0.37–1.16	0.58–0.59		
Time	<0.001	***	0.13	0.007	**	0.02	<0.001	***	0.01–3.80	0.35–0.37		
WS				<0.001	***	0.10	<0.001	***	3.73	0.48	2.80	4.66
MBg				<0.001	***	0.05	<0.001	***	−3.14	0.63	−4.38	−1.90

Note: ANOVA = analysis of variance. ANCOVA = analysis of covariance corrected by weight status and migration background. Ped = parental education. PPh = Parental physical activity. PET = parental education * time. PPT = parental physical activity * time. WS = weight status. MBg = migration background. B = unstandardized regression coefficient. SE B = standard error of the unstandardized regression coefficient. 95% CI =: 95% confidence interval. lb = lower bound. ub = upper bound. * = 0.01 ≤ *p*, ** = 0.001 ≤ *p* < 0.010, *** = *p* < 0.001.

**Table 3:** Weight status and migration background distributions separately for children with parental education group and parental physical activity group.

		Time Point										
Variable		Year 1		Year 2		Year 3		Year 4		MC	*p*	
OW, % (n)	1+ parent USE	10.3%	(19)	12.7%	(25)	12.8%	(26)	13.3%	(28)	29–69	<0.010	**
	2 parents LSE	21.2%	(25)	24.2%	(29)	26.8%	(34)	30.3%	(40)			
MBg, % (n)	1+ parent USE	20.6%	(46)							7	<0.001	***
	2 parents LSE	46.8%	(66)									
OW, % (n)	1+ parent RPA	11.5%	(30)	14.6%	(38)	16.9%	(44)	16.9%	(44)	24–64	0.285 < *p* < 0.763	
	2 parents NPA	12.6%	(14)	14.4%	(16)	14.4%	(16)	21.6%	(24)			
MBg, % (n)	1+ parent RPA	24.6%	(64)							2	< 0.001	***
	2 parents NPA	44.1%	(49)									

Notes: *p*-values according to χ² tests. MC = missing cases. OW = overweight group. MBg = migration background. UES = upper secondary education or above. LSE = lower secondary education or below. RPA = regular physical activity. NPA = irregular or no physical activity. ** = 0.001 ≤ *p* < 0.010, *** = *p* < 0.001.

## Data Availability

The data presented in this study are available on request from the corresponding author. The data are not publicly available due to privacy restrictions.
